# Anti-Metastatic Effects on Melanoma via Intravenous Administration of Anti-NF-κB siRNA Complexed with Functional Peptide-Modified Nano-Micelles

**DOI:** 10.3390/pharmaceutics12010064

**Published:** 2020-01-15

**Authors:** Hisako Ibaraki, Takanori Kanazawa, Minami Owada, Keiko Iwaya, Yuuki Takashima, Yasuo Seta

**Affiliations:** 1 School of Pharmacy, Tokyo University of Pharmacy and Life Sciences, 1432-1 Horinouchi, Hachioji, Tokyo 192-0392, Japan; ibaraki@toyaku.ac.jp (H.I.); m.owada@kobayashi.co.jp (M.O.); iwayakeiko@gmail.com (K.I.); takasima@toyaku.ac.jp (Y.T.); setayas@toyaku.ac.jp (Y.S.); 2 School of Pharmacy, Nihon University, 7-7-1 Narashinodai, Funabashi, Chiba 274-8555, Japan

**Keywords:** polymer nano-micelles, siRNA, systemic delivery, metastasis, melanoma, NF-κB, RelA, functional peptide

## Abstract

Controlling metastasis is an important strategy in cancer treatment. Nanotechnology and nucleic acids with novel modalities are promising regulators of cancer metastasis. We aimed to develop a small interfering RNA (siRNA) systemic delivery and anti-metastasis system using nanotechnology. We previously reported that polyethylene glycol-polycaprolactone (PEG-PCL) and functional peptide CH2R4H2C nano-micelle (MPEG-PCL-CH2R4H2C) has high siRNA silencing effects, indicated by increased drug accumulation in tumor-bearing mice, and has an anti-tumor effect on solid tumors upon systemic injection. In this study, we aimed to apply our micelles to inhibit metastasis and evaluated the inhibitory effect of anti-RelA siRNA (siRelA), which is a subunit of NF-κB conjugated with MPEG-PCL-CH2R4H2C, via systemic administration. We report that siRelA/MPEG-PCL-CH2R4H2C had a high cellular uptake and suppressed the migration/invasion of cells in B16F10 cells without toxicity. In addition, in a lung metastasis mouse model using intravenous administration of B16F10 cells treated with siRelA/MPEG-PCL-CH2R4H2C, the number of lung nodules in lung tissue significantly decreased compared to naked siRelA and siControl/MPEG-PCL-CH2R4H2C micelle treatments. Hence, we show that RelA expression can reduce cancer metastasis, and MPEG-PCL-CH2R4H2C is an effective siRNA carrier for anti-metastasis cancer therapies.

## 1. Introduction

Cancer causes millions of deaths worldwide, and the metastasis and invasion abilities of cancer cells especially increases the number and pathological condition of cancer patients [1]. Lung metastases are found in 20–54% of deceased cancer patients [2]. The metastatic spread of tumor cells from the primary site to other organs via blood or lymph vessels causes high mortality rates and remains a serious challenge for cancer treatment [3,4,5]. Organs prone to metastasis include the lung, liver, and brain. While there have been good advances in clinical non-invasive modalities such as immunotherapies like Nivolumab (Opdivo, Ono Pharmaceutical Co., Ltd., Osaka, Japan) or Ipilimumab (Yervoy, Bristol-Myers Squibb, New York, NY, USA), and other antibody-based medicines, the therapeutic efficacy and patient compatibility are quite limited and with some severe side effects [6,7]. Therefore, the epochal method of controlling metastasis has been the focus of biological and clinical cancer research [7,8,9] to develop new strategies that are urgently needed. Indeed, the low efficacy of current therapies on cancer metastasis is the result of poor drug delivery of current therapeutic agents to metastatic sites [10,11].

Breakthrough nanotherapeutics such as liposome or polymeric micelles hold great promise for improving the amount of the anti-cancer drugs that can reach metastatic sites by enhancing drug permeability and retention (EPR) [12,13,14,15]. A liposomal formulation (Onivyde: Merrimack Pharmaceuticals, Cambridge, MA, USA) for the treatment of metastatic pancreatic cancer was approved by the Food and Drug Association (FDA) in 2015 [16]. It is an irinotecan encapsulated liposome, which improves the pharmacokinetics by increasing drug encapsulation and loading efficiency, prolonging circulation time, rerouting the drug from sites of toxicity such as the gastrointestinal tract, and increasing drug accumulation via the EPR effect without toxicity [17,18].

Nucleic acids are great anti-cancer treatment candidates with a novel modality that can target molecules such as mRNA for cancer treatment. Although it is not part of the oncology field, ONPATTRO^®^ (Patisiran; Alnylam Pharmaceuticals Inc., Cambridge, MA, USA) was approved by the FDA in August 2018 as a novel combination of lipid nanoparticles and nucleic acid (small interfering RNA; siRNA) [19]. Therefore, expectations for nanotherapeutics are increasing.

Methoxy-polyethylene glycol combined polycaprolactone conjugated with a cytoplasm-responsive peptide CH2R4H2C (MPEG-PCL-CH2R4H2C) has been previously confirmed to form micelles with a particle size of 100 nm or less and has high drug accumulation in tumor-bearing mice [20,21]. Furthermore, the suppression of solid tumor growth by intravenous administration using siRNA against vascular endothelial cell growth factor (VEGF) with MPEG-PCL-CH2R4H2C was also reported [20]. Hence, we aimed to trial our developed polymeric micelle as a novel nanocarrier for siRNA systemic delivery that can both control pharmacokinetics and intracellular trafficking using the di-block copolymer, MPEG-PCL-CH2R4H2C.

Hematogenous metastasis occurs when cancer cells separate from the primary tumor site, invade surrounding tissues like inter-stroma and migrate through blood vessels, and then adhere to vascular endothelial cells. Initial adhesion occurs by cellular rolling on vascular endothelial cells and then adhere to those cells by integrin, intercellular cell adhesion molecule (ICAM) and vascular cell adhesion molecule (VCAM) [22]. In this study, we focused on RelA as a target molecule for inhibiting cancer metastasis because it is a subunit of NF-κB that was recently reported to be involved in cancer metastasis, especially migration and invasion [23,24]. In particular, we focused on suppressing migration to anti-metastasis because metastasis would not spread without migration. NF-κB consists of two subunits that form homo- or hetero-dimers, with the most typical complex consisting of p50 and RelA (p65). RelA is normally present in the cytoplasm and exists as a complex with IκB, but in response to inflammatory signals from extracellular cytokines, IκB is phosphorylated and NF-κB is translocated into the nucleus by a nuclear translocation signal, where it exhibits transcriptional activity and controls the expression of cell adhesion molecules E-selectin, ICAM, and VCAM [25,26].

NF-κB is a central inflammatory mediator and regulates multiple proinflammatory genes in allergic diseases [27,28]. Recent studies demonstrate that RelA is also involved in tumor angiogenesis in various malignances. Increases in NF-κB activity due to cancer or inflammation can also increase production of proteins associated in neovascularization of tumor blood vessels such as VEGF, monocyte chemoattractant factor-1, interleukin-8, and cyclooxygenase-2. Moreover, the suppression of NF-κB activity is thought to be anti-growth and anti-metastatic for some cancers. Indeed, the inhibition of RelA expression with RelA siRNA enhanced in vitro and in vivo colon cancer cell responses to irinotecan [29,30]. These results indicate that RelA could offer another potential therapeutic strategy for controlling metastasis [31].

The inhibitory efficiency of RelA with CH2R4H2C for treatment of allergy such as atopic dermatitis or collagen-induced arthritis mice was previously reported [21,32,33,34,35]. The carrier with CH2R4H2C is a revolutionary system that exhibits high siRNA delivery ability to inflammatory sites and has a remarkable RelA silencing effect. Based on these findings, we hypothesized that siRelA with MPEG-PCL-CH2R4H2C could inhibit cell-adhesion between migrating cancer cells and vascular endothelial cells by inhibiting the expression of RelA through gene silencing with siRelA, and therefore improve tumor accumulation and the silencing effect of MPEG-PCL-CH2R4H2C through systemic administration.

In this study, we evaluated the effect of siRelA/MPEG-PCL-CH2R4H2C on the metastatic potential of the highly metastatic malignant tumor cells line, murine melanoma B16F10 in an in vitro system. Moreover, we investigated whether siRelA/MPEG-PCL-CH2R4H2C administration, which is the nano-system used to transfect siRNA into cells in situ and inhibited the pulmonary metastasis of B16F10 melanoma after intravenous injection in mice.

## 2. Materials and Methods

### 2.1. Materials, Cells, and Mice

The anti-RelA siRNA (siRelA; sense: 5’-GGUGCAGAAAGAAGACAUU-3’), Control siRNA (siControl; 5’-AUCCGCGCGAUAGUACGUA-3’) and Fluorescence 6-carboxyfluorescein-aminohexylphosphoramidite (FAM)-labeled siRNA (FAM-siRNA; sense: 5’-6FAM-AUCCGCGCGAUAGUACGUA-3’) were purchased from Nippon Gene Co., Ltd. (Tokyo, Japan). Methoxy polyethylene glycol (Mn: 2000)-polycaprolactone (Mn: 2000) (MPEG-PCL) was obtain from Taki Chemical Co., Ltd., Hyogo, Japan), CH2R4H2C peptide (Cys-His-His-Arg-Arg-Arg-Arg-His-His-Cys) was obtained from BEX Co., Ltd. (Tokyo, Japan). Dimethyl formamide (FUJIFILM Wako Pure Chemical Corporation, Osako, Japan), *N*-(3-Dimethyl aminopropyl)-*N*’-ethyl carbodiimide 4-Dimethyl aminopyridine (Sigma-Aldrich Co., Milwaukee, WI, USA) were used for synthesis of MPEG-CH2R4H2C. The mice melanoma B16F10 cell line and rat retinal pigment epithelial RPE-J cell lines were purchased from American Type Culture Collection (Manassas, VA, USA). Dulbecco’s modified Eagle medium (DMEM), certified fetal bovine serum (FBS), penicillin/streptomycin stock solutions, 0.25% Trypsin-EDTA, and minimum essential medium non-essential amino acid solution (NEAA) were purchased from Thermo Fisher Scientific, Waltham, MA, USA. Lipotrust™ (Hokkaido System Science, Sapporo, Japan), cell counting kit-8 (CCK-8, Dojindo Laboratories, Kumamoto, Japan) solution and dimethyl sulfoxide (DMSO; Wako Pure Chemical Corporation, Osaka, Japan) were used for the cellular uptake and cytotoxicity assays in B16F10 cells. Additionally, Mitomycin C (Sigma-Aldrich Co., Milwaukee, WI, USA), and Bouin’s solution (Sigma-Aldrich Co., Milwaukee, WI, USA) were used. All animal experiments were carried out in accordance with a protocol approved by the Animal Care and Ethics Committee of Tokyo University of Pharmacy and Life Sciences. The project identification code was P14-03 (8 May 2014), P15-67 (7 May 2015). The mice were housed under the standard conditions, temperature was 23.5 ± 1 °C, humidity was 55% ± 5%, and light/dark-cycles every 12 h and food and water were supplied ad libitum.

### 2.2. Synthesis of CH2R4H2C Conjugated to MPEG-PCL

The MPEG-PCL-CH2R4H2C was synthesized and the conjugation reaction was confirmed as previously described [20,21]. The ratio and volume between MPEG-PCL and CH2R4H2C were at optimal values after careful consideration. MPEG-PCL and CH2R4H2C were dissolved in dimethylformamide (DMF) and then water soluble carbodiimide hydrochloride (WSCI) were added to this mixture and allowed to react at room temperature for 24 h to cause the Cys-COOH on the C-terminus of CH2R4H2C and the terminal –OH group on MPEG-PCL to form an ester bond. The reaction solution was transferred to a dialysis membrane suitable for use with organic solvents (Spectra/Por^®^ Dialysis Membranes, MWCO: 3500) and dialyzed against distilled water for 3 days. Afterwards, the product was lyophilized to yield MPEG-PCL-CH2R4H2C.

### 2.3. Preparation of siRNA/MPEG-PCL-CH2R4H2C

First, each siRNA and MPEG-PCL-CH2R4H2C solution was prepared using RNase free water. Each solution was mixed at a nitrogen/phosphorous (N/P) molar ratio of 5, 10, 15, 20 and 30, and incubated for 30 min at 20 ± 5 °C before use. The siRNA/MPEG-PCL-CH2R4H2C complexes were mixed in equal quantities.

The physical properties (particle size, zeta (Z) potential, and poly-dispersity index (PDI) of siRNA/MPEG-PCL-CH2R4H2C micelles were measured with a Zetasizer Nano (Malvern P analytical, Malvern, UK). The particle sizes were obtained by measuring the average particle size using the dynamic light scattering method and calculating the volume average size as the particle size distribution. Z potential was measured by electrophoretic light scattering. The results indicate the average data of three experiments.

### 2.4. Evaluation of siRNA Cellular Uptake Ability of siRNA/MPEG-PCL-CH2R4H2C

The B16F10 cells were pre-cultured to about 80% confluence in DMEM containing 10% fetal bovine serum (FBS) and 1% penicillin/streptomycin before in vitro transfection. Then, 2 × 10^5^ B16F10 cells were seeded into 6-well culture plates and cultured at 37 °C in a humidified 5% CO_2_ atmosphere. After 24 h incubation in DMEM containing 10% FBS, the cells were washed with phosphate buffered saline (PBS) and fresh DMEM was added before transfection with naked FAM-siRNA, or siRNA/MPEG-PCL-CH2R4H2C (N/P 5, 10, 20, and 30). The N/P ratio is the molar ratio of arginine-derived N in the CH2R4H2C peptide to the phosphate group of siRNAs, and the cationic charge increases with the N/P ratio. The siRNA concentration used for transfection was 1 µg/100 µL. After 24 h incubation, the culture medium was aspirated, and the cells were washed with PBS. Cells were detached by pipetting and resuspended in PBS and then analyzed using flow cytometry (BD FACS Canto, Maywood, NJ, USA). Cell number was plotted relative to fluorescence intensity in the control of the target cell group; this indicated that the range including 95% of the cells was defined as the P1 region (10,000 cells) and the range in which the fluorescence intensity was stronger was defined as the P2 region. Thus, the percentage of cells in the P2 region was used as an indicator of siRNA uptake into the cells (control group was set to 5% in the P2 region). Additionally, the fluorescence intensity per cell was calculated by the average value of fluorescence intensity in the P2 region as the cellular uptake efficiency.

### 2.5. Evaluation of the Cytotoxicity of MPEG-PCL-CH2R4H2C on Normal Cells

RPE-J cells (6 × 10^3^ cells/well) in 100 μL DMEM containing 10% FBS and 1% NEAA were seeded into 96-well plates and incubated at 37 °C in a humidified 5% CO_2_ atmosphere. After 24 h, the cells were washed with PBS and transfected with naked-siControl and siControl/MPEG-PCL-CH2R4H2C (N/P 5, 10, 15, 20, or 30; 100 nM of siRNA). After transfection for 4 h, CCK-8 solution (Dojindo laboratories, Kumamoto, Japan) was added to each well and the cells were incubated for an additional 3 h. The absorbance of the cells in each well was measured at 450 nm using a microplate reader (Varioskan Flash 2.4, Thermo Fisher Scientific., MA, USA). The absorbance of the control cells was set as the 100% viability standard.

### 2.6. Cytocidal Assay for siRelA/MPEG-PCL-CH2R4H2C

B16F10 cells (6 × 10^3^ cells/well) in 100 μL DMEM containing 10% FBS were seeded into 96-well plates and incubated at 37 °C in a humidified 5% CO_2_ atmosphere. After 24 h, the cells were washed with PBS and transfected with siControl/MPEG-PCL-CH2R4H2C (N/P ratio; 10, siRNA concentration; 10, 50, 100 and 300 nM). After 4 h of transfection and 20 h incubation at 37 °C in a humidified 5% CO_2_ atmosphere, the same method for the cytotoxicity assay described in Section 2.5 was followed.

### 2.7. Wound Healing Migration Assay

B16F10 cells (4 × 10^5^ cells/well) suspended in 2 mL DMEM containing 10% FBS were seeded into 6-well plates and incubated at 37 °C in a humidified 5% CO_2_ atmosphere. After 24 h, Mitomycin C (25 μg/mL) was added to the cells and incubated for 30 min [36]. After washing with DMEM, lines (width: 2 mm) were drawn through a confluent monolayer of cells and naked-siRelA, siControl/MPEG-PCL-CH2R4H2C and siRelA/MPEG-PCL-CH2R4H2C (N/P ratio 10) were transfected for 4 h at an siRNA concentration of 100 nM. The medium was aspirated and 2 mL DMEM was added to each well before the cells were incubated for 68 h. The cells were washed and fixed by 4% paraformaldehyde for 10 min. These samples of scratched area were observed by an optimal microscope (BZ8000, KEYENCE, Osaka, Japan) and analyzed by ImageJ software version 1.45 (https://imagej.nih.gov/ij/index.html).

### 2.8. In Vivo Experimental Pulmonary Metastasis Assay

Six-week-old male C57BL/6 mice were divided into three groups (*n* = 4 for each group) and B16F10 cells (1.5 × 10^5^ cells/100 µL) were injected into the tail veins using a 27G needle. The mice were injected with saline (Control), naked-siRelA, siControl/MPEG-PCL-CH2R4H2C and siRelA/MPEG-PCL-CH2R4H2C (N/P ratio: 10, siRNA Dose: 20 µg) on days 0, 1, 2, 4, 6, 8 and 10. The mice were sacrificed on day 14 and the lungs were harvested. Lungs were fixed in Bouin’s solution 30 min and the number of B16F10 nodules on the surface of the front and back of the lungs were determined by visual inspection and imaged for further analysis.

### 2.9. Statistical Analysis

The statistical analysis was performed by BellCurve for Excel (Social Survey Research Information Co., Ltd., Tokyo, Japan). The results of the experiments are represented as the mean ± S.D. Comparisons between multiple treatments were made using analysis of variance (ANOVA), followed by Dunnett’s test. Group differences were considered statistically significant when *p* < 0.05. Statistical significance was defined as * *p* < 0.05 and ** *p* < 0.01.

## 3. Results

### 3.1. Physical Properties, Cellular Uptake and Cytotoxicity of siRNA/MPEG-PCL-CH2R4H2C

The physical properties of siRNA/MPEG-PCL-CH2R4H2C were demonstrated to have a particle size of approximately 50 nm and were positively charged at an N/P ratio of 10, as in shown Table 1. When the N/P ratio increased, the particle size decreased and the Z-potential increased. PDI, which is an indication of their quality with respect to size distribution was approximately 0.4 in any N/P ratio.

**Figure 1:**
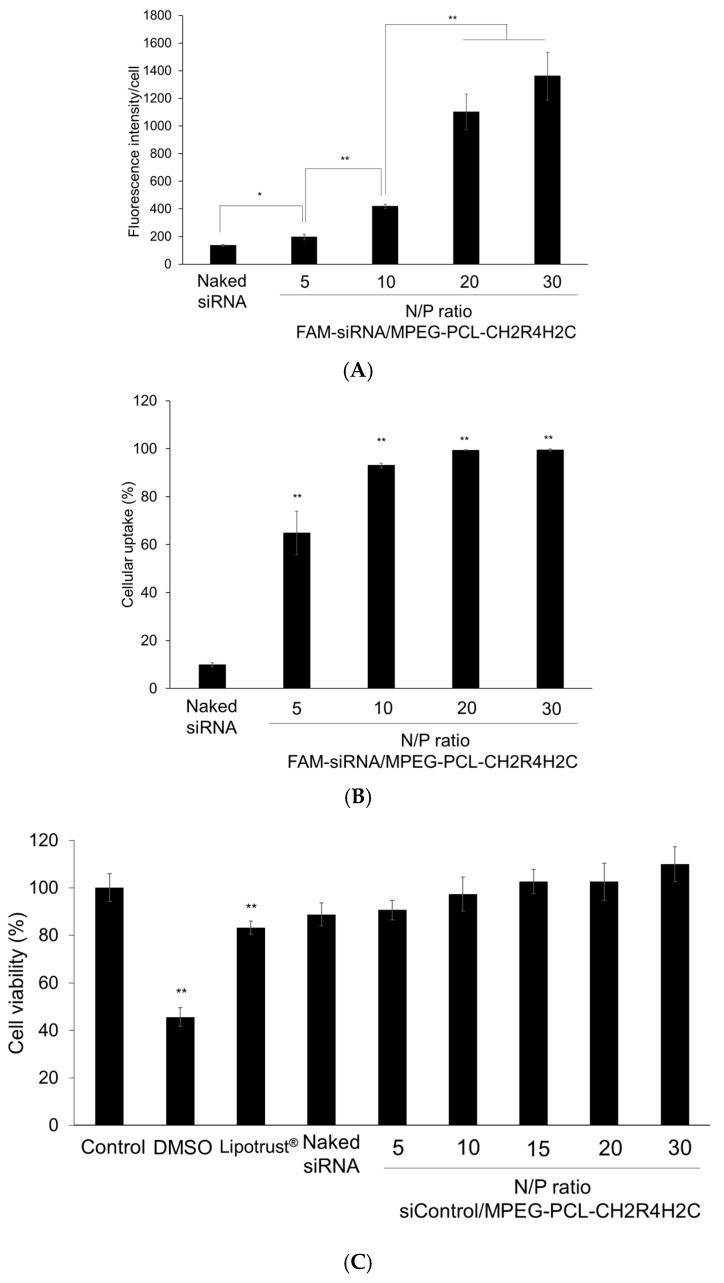
Cellular uptake ability and cytotoxicity of the siRNA/MPEG-PCL-CH2R4H2C. (**A**) siRNA cellular uptake (%) compared to the control group. (**B**) Fluorescent intensity per cell of FAM-siRNA in B16F10 cells. Naked FAM-siRNA or FAM-siRNA with MPEG-PCL-CH2R4H2C (N/P ratio; 5, 10, 20 and 30, siRNA concentration: 1 µg/100 µL) was transfected into B16F10 cells for 24 h and each sample was measured by flow cytometry. Each bar represents the mean ± S.D. (*n* = 3) * *p* > 0.05, ** *p* > 0.01 vs. naked siRNA group. (**C**) Cytotoxicity of MPEG-PCL-CH2R4H2C in RPE-J cells. Naked siControl and siControl with MPEG-PCL-CH2R4H2C (N/P ratio: 5, 10, 15, 20 and 30, siRNA concentration: 100 nM) were transfected into B16F10 cells for 4 h without serum. Cell viability was evaluated with a CCK-8 assay. The control value was taken as 100% and calculated as a relative value. Each bar represents the mean ± S.D. (*n* = 5–8). ** *p* > 0.01 vs. Control group.

The cellular uptake ability of siRNA/MPEG-PCL-CH2R4H2C micelles by B16F10 cells was evaluated with flow cytometry as shown in Figure 1A. The siRNA cellular uptake percentage (%) indicated a high uptake ability that was above 90% compared to naked siRNA groups. We also confirmed that MPEG-PCL-CH2R4H2C micelles with an N/P ratio of five or more had significantly improved cellular uptake efficiency. In addition, the fluorescence intensity per cell increased in relation to the N/P ratio; the fluorescence of micelles with an N/P five was not much different from naked siRNA, whereas those with an N/P 10 had about five times the fluorescence of naked siRNA as shown in Figure 1B. Micelles with an N/P 20 had an uptake efficiency that was more than 10 times that of naked siRNA. Therefore, the fluorescence intensity per cell increased as the N/P ratio increased and an increase in the N/P ratio indicated an increase in the ratio of MPEG-PCL-CH2R4H2C to siRNA addition, Figure 1C shows the cytotoxicity of MPEG-PCL-CH2R4H2C itself using siControl with the CCK-8 assay, which is a sensitive colorimetric assay method for the determination of cell viability. The rat retinal pigment epithelial cell, RPE-J, was used as the normal cell to evaluate cytotoxicity. Additionally, DMSO was used as the negative control because of its high cytotoxicity. As a result, the high cytotoxicity of DMSO, and Lipotrust^®^ which is a commercially available transfection reagent, was demonstrated confirmed compared to the control group. On the other hand, the cytotoxicity of MPEG-PCL-CH2R4H2C itself was never observed at any N/P ratio in normal cells.

### 3.2. Cell Migration Suppression of siRelA/MPEG-PCL-CH2R4H2C

To investigate the effect of siRelA/MPEG-PCL-CH2R4H2C on metastatic activity, the migration activity of B16F10 cells was assessed at 0 and 72 h with a wound healing assay. This method can semi-quantitatively measure cell migration for specifically evaluating cell migration in an in vitro monolayer cell culture system. If cells can migrate after wounding, the area scratched and treated with mitomycin-C is covered with cells over time; whereas if cell migration is suppressed, the treated area is not repopulated. Besides, the N/P ratio of siRNA/MPEG-PCL-CH2R4H2C was selected as 10 based on the above results. As the N/P ratio increases, the amount of MPEG-PCL-CH2R4H2C increases. It was thought the N/P 10 was as little a carrier amount as possible while still showing the function. Moreover, we have previously confirmed that the siRNA/MPEG-PCL-CH2R4H2C could be condensed at N/P ratios above 5 [20,21].

**Figure 2:**
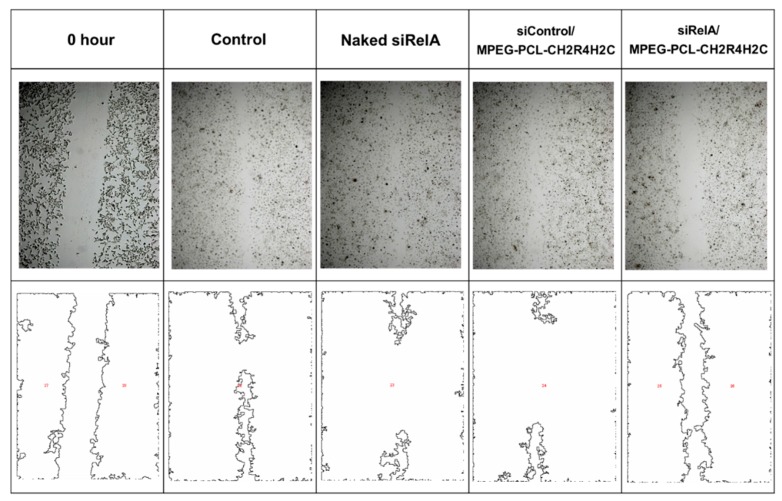
In vitro wound healing assay of B16F10 cells expressing siRelA/MPEG-PCL-CH2R4H2C. B16F10 cells were seeded onto 6-well plates and incubated for 24 h. Mitomycin C (25 μg/mL) was added to the cultures and lines were scratched (width: 2 mm) into the confluent cell monolayer for 30 h. Naked siRelA, siControl/MPEG-PCL-CH2R4H2C, and siRelA/MPEG-PCL-CH2R4H2C (N/P ratio; 10, siRNA concentration; 100 nM). After 72 h, these samples were fixed in 4% paraformaldehyde for 10 min, observed with optical microscope and analyzed with ImageJ software. Upper panel: bright field image; Bottom panel: example of a representative image in ImageJ used for analysis.

Figure 2 shows the wound area, 72 h after wounding, was imaged and analyzed using ImageJ. It showed that in the control, naked siRelA, and siControl/MPEG-PCL-CH2R4H2C, the wounded area width was reduced from the initial wound width. Conversely, cells transformed with siRelA/MPEG-PCL-CH2R4H2C clearly showed no cell migration into the wound area. These results indicate that siRelA/MPEG-PCL-CH2R4H2C had anti-migration activity.

### 3.3. Cytocidal Activities of siRelA/MPEG-PCL-CH2R4H2C

**Figure 3:**
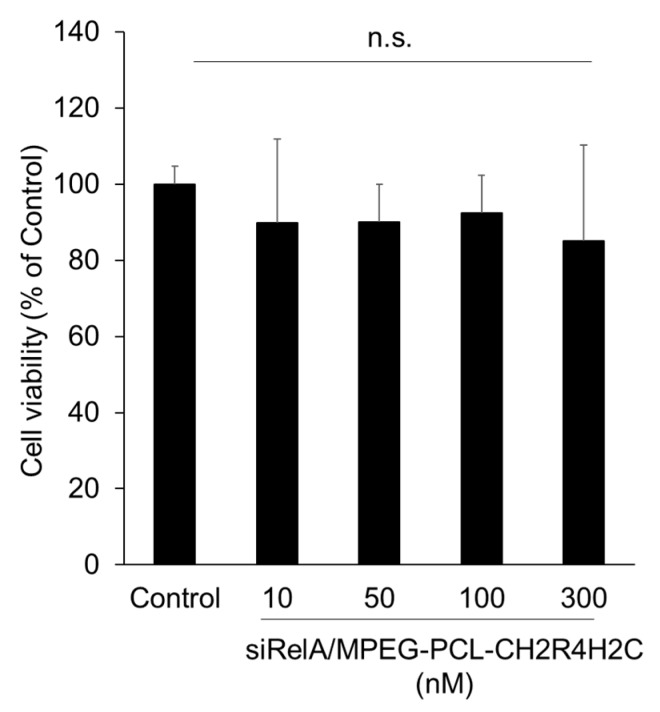
In vitro cytocidal activity of siRelA/MPEG-PCL-CH2R4H2C in B16F10 cells. Naked siRelA and siRelA/MPEG-PCL-CH2R4H2C (N/P ratio: 10, siRNA concentration: 10, 50, 100, 200 and 300 nM) were transfected into B16F10 cells for 24 h without serum. The cell viability was evaluated with a CCK-8 assay. The control value was taken as 100% and calculated as a relative value. Each bar represents the mean ± S.D. (*n* = 3). ^n.s^. *p* < 0.05.

Figure 3 shows the cytocidal activity of siRelA/MPEG-PCL-CH2R4H2C in B16F10 cells evaluated with a CCK-8 assay. Unlike in the cytotoxicity evaluation, the difference between the results of the cytotoxicity (siControl) and cytocidal activity (siRelA) experiments indicated the effect of siRelA because siRelA was used for this cytocidal evaluation. The cell viability of siRelA/MPEG-PCL-CH2R4H2C micelles did not decrease compared to the control, after 24 h even at 300 nM, which was a high concentration. These results indicate that siRelA/MPEG-PCL-CH2R4H2C had anti-migration activity without cytocidal activity.

### 3.4. In Vivo Anti-Metastasis Activity by Intravenous Administration of siRelA/MPEG-PCL-CH2R4H2C

**Figure 4:**
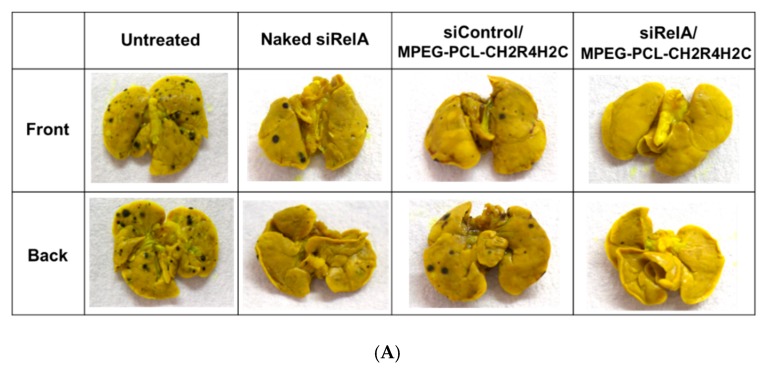

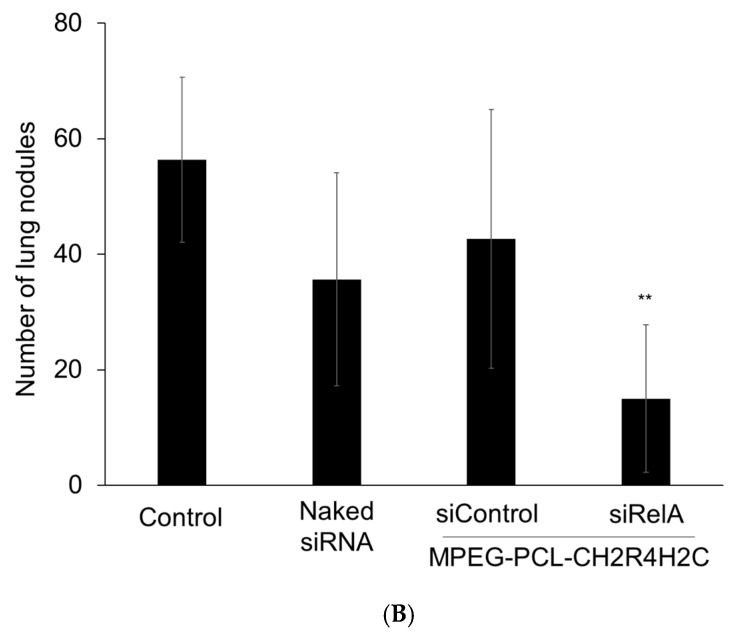
Anti-metastatic effects of intravenous administration of B16F10 cells expressing siRelA/MPEG-PCL-CH2R4H2C in a lung metastatic mouse model Saline (Control), naked siRelA, siControl/MPEG-PCL-CH2R4H2C and siRelA/MPEG-PCL-CH2R4H2C (N/P ratio:10, siRNA Dose: 20 µg) at on days 0, 1, 2, 4, 6, 8 and 10. The mice were sacrificed at day 14 and the lungs were removed. Lungs were (**A**) fixed in Bouin’s solution for and (**B**) the number of B16F10 nodules on the surfaces of the front and back sides of the lungs were imaged and quantified. Each bar represents the mean ± S.D. (*n* = 4) ** *p* > 0.01 vs. control group.

To explore the anti-metastatic effects of our siRNA delivery system, a lung metastasis model was prepared by intravenously administering B16F10 cells to six-week-old male C57BL/6 mice and siRNA agents by seven times, and then the number of lung metastasis nodules was measured on the 14th day after treatment (Figure 4). This model is a well-established metastasis model that spontaneously develops lung metastasis after intravenous administration of B16F10 cells [37,38,39,40]. In addition, the survival rate of animals in this experimental was 100% and there were no weakened mice; thus, this metastasis mice model was an appropriate method.

As shown in Figure 4, several lung nodules (e.g., black colonies) of B16F10 were observed on the lung surface and inside in the untreated, siControl/MPEG-PCL-CH2R4H2C and naked siRelA groups. However, metastatic nodules were significantly reduced in lungs from mice treated with seven systemic injections of siRelA/MPEG-PCL-CH2R4H2C. Moreover, the number of nodules in mice treated with siRelA/MPEG-PCL-CH2R4H2C was fewer than the other treatment groups.

## 4. Discussion

Controlling metastasis is one of the most important strategies in cancer treatment. Once cancer has metastasized, it is difficult to suppress and is responsible for about 50% of cancer patient mortalities [2]. Chemotherapy after metastasis has been reported to improve therapy efficiency in reducing liver metastasis in colorectal cancer; however, cancers can re-metastasize several times even after surgical resection, with significantly reduced 5-year survival rates and quality of life (QOL) of patients [12]. Hence, novel treatment systems that can suppress cancer metastasis are desired by both the clinical and public communities.

In this study, we aimed to develop a novel anti-metastasis system using a new modality for siRNA and functional peptide-modified polymeric micelles and evaluated its anti-metastasis effect on melanoma. Intractable metastatic cancer is one of the deadliest diseases. Additionally, melanoma is one of the most metastatic forms of cancer. NF-κB affects cancer invasion and migration [21,24,25,41]; hence, RelA of the NF-κB subunit was selected as the molecular target for metastasis for testing this new system.

**Scheme 1:**
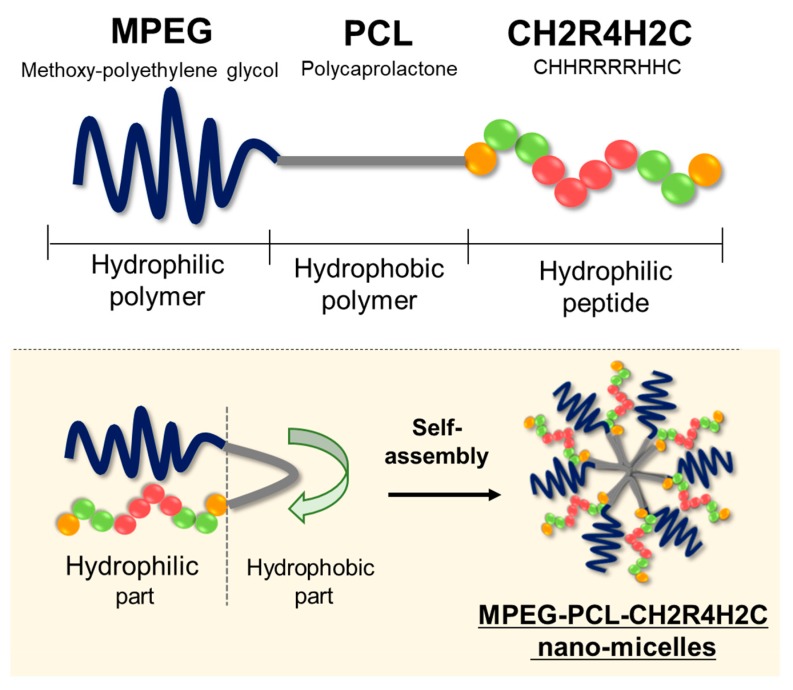
The structure of MPEG-PCL-CH2R4H2C.

First of all, the siRelA/MPEG-PCL-CH2R4H2C complex was prepared, and its physical properties were evaluated. siRelA and MPEG-PCL-CH2R4H2C could form stable complex having particle size of approximately 50 nm and positively charged at an N/P ratio of 10 (Table 1). Moreover, PDI values were approximately 0.4. PDI values of more than 0.7 show a broad particle size distribution. A PDI value of 0.05 or less is considered to be the ideal mono-dispersion. However, a PDI value of 0.4 may not be as good regarding dispersibility. This suggests that further improvement is needed. We hypothesized that our MPEG-PCL-CH2R4H2C micelles form a “V” shape spontaneously that would expose hydrophilic PEG chains and CH2R4H2C on the surface of the micelle, and that the hydrophobic PCL would fold to allow each moiety to exert its respective function (Scheme 1). It was proposed that a hydrophilic–hydrophobic–hydrophilic tri-block polymer micelle also forms “V”-shaped polymers that confer a high silencing effect on the micelles and siRNA complexes both in vitro and in vivo [42,43]. In terms of functionality, a PEG chain with MPEG-PCL is expected to improve blood retention and tumor accumulation due to the EPR effect, and CH2R4H2C would further improve intracellular dynamics as our previous studies [44,45]. Generally, arginine (R) forms a complex by electrostatic interaction with siRNA, which improves intracellular ability and helps the siRNA escape endosomal degradation by the histidine (H) proton sponge effect. Moreover, cysteine (C) releases siRNA through the cleavage of disulfide bonds in the cytoplasm. We have reported that the MEPG-PCL-peptide and CH2R4H2C are useful for many intractable diseases for siRNA delivery [20,21,32,33,34,35,44,45,46,47], and we tried to apply this method of therapeutic delivery to metastatic cancer in the present study. Regarding PCL, MPEG-CH2R4H2C can act as a nucleic acid carrier, but micellization does not occur without PCL, which is a hydrophobic part. Additionally, the micelles do not stabilize and can be large because the internal structure cannot be made. Therefore, we believe that PCL is indispensable for the preparation of the stable micelles that can be administered intravenously with a particle size of 100 nm or less.

Additionally, we confirmed that MPEG-PCL-CH2R4H2C micelles with an N/P ratio 5 or more had significantly improved the siRNA cellular uptake efficiency in melanoma B16F10 cells (Figure 1A). In particular, the complex with a N/P ratio of more than 10 had about above five times that of naked siRNA (Figure 1B). The fluorescence intensity per cell indicated the ability for carrier uptake directly into cells. Therefore, the cellular uptake ability increased as the N/P ratio increased and an increase in the N/P ratio indicated an increase in the ratio of MPEG-PCL-CH2R4H2C to siRNA. The intracellular uptake efficiency is improved by increasing the N/P ratio because of the contribution of the guanidino group in arginine. In a future study, qualitative evaluation using confocal laser microscopy will be evaluated. Then, the cytotoxicity of MPEG-PCL-CH2R4H2C itself was never observed at any N/P ratio, even at an N/P ratio of 30, in normal cells (RPE-J) (Figure 1C). Therefore, these results suggest that MPEG-PCL-CH2R4H2C is a safe and effective siRNA carrier.

Next, we investigated the effect of siRelA/MPEG-PCL-CH2R4H2C on cell migration activity of B16F10 metastatic cells assessed with a wound healing assay. Cancer metastasis occurs in the order of invasion, migration, circulation, and extravasation. Among these stages, migration is considered to be the most important factor that determines the prognosis of metastasis. As migration is associated with RelA expression, the suppression of RelA is crucial for the suppression of metastasis and measuring the degree of wound healing is a useful method to visualize the degree of migration. Besides, the N/P ratio of siRNA/MPEG-PCL-CH2R4H2C was selected as 10 based on the above results. As shown in Figure 2, it was confirmed that cells transformed with siRelA/MPEG-PCL-CH2R4H2C clearly showed no cell migration into the wound area Conversely, the wounded area width was reduced from the initial wound width in the control, naked siRelA, and siControl/MPEG-PCL-CH2R4H2C. These results indicate that siRelA/MPEG-PCL-CH2R4H2C had anti-migration activity of melanoma. At this time, the cell viability of siRelA/MPEG-PCL-CH2R4H2C group did not decrease, unlike that in the control group, after 24 h even at 300 nM, which was a high concentration (Figure 3). In other words, suppression of RelA did not directly kill cancer cells, but suppressed metastasis/invasion. Furthermore, the effects of siRNA were improved by the high intracellular introduction efficiency of the MEPG-PCL-CH2R4H2C micelles. Moreover, it suggested that RelA made a significant contribution to cancer cell migration and invasion by suppressing the expression of cell adhesion factors like ICAM.

Finally, we explored the anti-metastatic effects of our siRNA delivery system in vivo using a lung metastasis model prepared by intravenously administering B16F10 cells and siRNA agents seven times. The intravenous administration of cancer cells and the injection of cancer cells into the spleen for liver metastasis are well-established methods to prepare an animal model of hematogenous metastasis, which is one of the major routes of cancer metastasis. Additionally, the survival rate of animals in this study was 100% and no mice were weakened, suggesting that this was a sufficiently safe experimental method. As shown in Figure 4, metastatic nodules were significantly reduced in lungs from mice treated with seven systemic injections of siRelA/MPEG-PCL-CH2R4H2C compared to that in the untreated group and was fewer than the other treatment groups on the 14th day after treatment. In general, NF-κB regulates the expression of genes involved in many processes that play key roles in the development and progression of cancer such as proliferation, migration and apoptosis. Aberrant or constitutive NF-κB activation has been detected in many human malignancies [21,24,25,48]. Treatment with siRelA/MPEG-PCL-CH2R4H2C significantly decreased the number of lung nodules, which were quantified by measuring visible nodules (Figure 4) and while this provides evidence of anti-metastatic effects, this result should be further validated. The administration of siRNA alone proved difficult to show that the effect is due to degradation and low bioavailability [49,50]. Therefore, siRNA carriers are indispensable. According to a report of nanotherapeutics for the treatment of metastasis, poly-ion complex micelles composed of PLL and PEG with a particle size of 30 nm encapsulated in dahaplatin penetrated blood vessels in tumors that had spread to lymph nodes can penetrate deep into the metastasis [13]. In addition, there are cases where tumor metastasis can be suppressed by intravenous administration of liposomal nanoparticles carrying the tumor-homing pentapeptide CREKA without side effects [51]. MPEG-PCL-CH2R4H2C was more effective than naked siRelA and improved siRNA delivery to the metastatic site; therefore, MPEG-PCL-CH2R4H2C indicated as a useful in vivo anti-metastatic siRNA delivery system. Moreover, although the pharmacokinetics was improved by PEG modification to the drug carrier, PEG is a hindrance for intracellular kinetics, which is known as the “PEG dilemma” [52,53]. Our micelles did not appear to have this behavior and may be useful for pharmacokinetics and intracellular kinetics. In addition, we have previously reported the silencing effect of RelA mRNA level at the lesioned site after intravenous administration of siRelA/MPEG-PCL-CH2R4H2C by RT-qPCR method and demonstrated a remarkable inhibitory effect on the mRNA expression [21]. Therefore, in this study, we suggested that MPEG-PCL-CH2R4H2C worked as a useful siRNA carrier which has silencing ability in vivo and actually showed a metastasis inhibitory effect compared with naked siRelA and siControl/MPEG-PCL-CH2R4H2C, indicating that migration and invasion could be suppressed by RelA inhibition. In the future, we must demonstrate the silencing effect of siRelA/MPEG-PCL-CH2R4H2C on RelA expression in vitro and in vitro by qPCR, as we believe this will support the results in this study.

Based on these results, we suggest that the intravenous administration of MPEG-PCL-CH2R4H2C enhances siRelA delivery to the lung and enhances anti-metastatic activity.

## 5. Conclusions

MPEG-PCL-CH2R4H2C suppressed a cell migration/invasion effect in melanoma B16F10 cells. In vivo intravenous administration of siRelA/MPEG-PCL-CH2R4H2C micelles in a lung metastasis mouse model increased MPEG blood retention and accumulation at metastatic sites, as well as increased siRNA introduction into cancer cells. The inhibition of RelA affected the expression of cell adhesion factors, cell migration and invasion, and significant suppression of metastasis compared to the control conditions. From now on, measurement of RelA mRNA or protein level will be needed to elucidate more detailed effect and mechanism of this nano-micelle. Based on these experiments, siRelA/MPEG-PCL-CH2R4H2C micelles are expected to be effective in the suppression of cancer metastasis and we are confident that they are useful as novel siRNA carriers for cancer treatment. These findings may contribute to the clinical care of many cancer patients.

## Figures and Tables

**Table 1:** The mean diameter and zeta potential of siRelA/MPEG-PCL-CH2R4H2C.

N/P Ratio	Particle Size (nm)	Zeta Potential (mV)	PDI
5	64.5 ± 2.7	−16.7 ± 3.8	0.469 ± 0.055
10	51.2 ± 1.3	+5.0 ± 0.5	0.473 ± 0.073
15	53.0 ± 5.5	+13.3 ± 1.3	0.427 ± 0.014
20	49.8 ± 2.3	+18.6 ± 1.9	0.431 ± 0.011
